# Risk Factors for Recurrence of Community-Onset Urinary Tract Infections Caused by Extended-Spectrum Cephalosporin-Resistant Enterobacterales

**DOI:** 10.1093/ofid/ofad561

**Published:** 2023-11-08

**Authors:** Helen L Zhang, Reinaldo Perez, Jay Krishnan, Ebbing Lautenbach, Deverick J Anderson

**Affiliations:** Duke Center for Antimicrobial Stewardship and Infection Prevention, Division of Infectious Diseases, Duke University Medical Center, Durham, North Carolina, USA; Duke Center for Antimicrobial Stewardship and Infection Prevention, Division of Infectious Diseases, Duke University Medical Center, Durham, North Carolina, USA; Duke Center for Antimicrobial Stewardship and Infection Prevention, Division of Infectious Diseases, Duke University Medical Center, Durham, North Carolina, USA; Center for Clinical Epidemiology and Biostatistics, University of Pennsylvania Perelman School of Medicine, Philadelphia, Pennsylvania, USA; Duke Center for Antimicrobial Stewardship and Infection Prevention, Division of Infectious Diseases, Duke University Medical Center, Durham, North Carolina, USA

**Keywords:** drug-resistant urinary tract infection, extended-spectrum cephalosporin resistance, recurrent urinary tract infection, urinary tract infection, UTI

## Abstract

**Background:**

Extended-spectrum cephalosporin-resistant Enterobacterales (ESCrE) are an increasingly important cause of community-onset urinary tract infections (UTIs), including recurrent infections. We evaluated risk factors for recurrence among patients with community-onset ESCrE UTI.

**Methods:**

This retrospective cohort study included adults with community-onset ESCrE UTI in the Duke University Health System from April 2018 through December 2021. ESCrE UTI recurrence by the same species was assessed 14–180 days (ie, 6 months) after completion of antibiotic treatment. We evaluated the relationships between candidate risk factors and time to recurrence using Cox proportional hazards regression models.

**Results:**

Among 1347 patients with community-onset ESCrE UTI, 202 (15.0%) experienced recurrent infection during the 6-month follow-up period. Independent risk factors for recurrence included neurogenic bladder (adjusted hazard ratio [aHR], 1.8 [95% confidence interval {CI}, 1.2–2.6]; *P* = .005), prior history of UTI (aHR, 2.4 [95% CI, 1.7–3.3]; *P* < .001), and fluoroquinolone nonsusceptibility of the index UTI (aHR, 1.5 [95% CI, 1.1–2.1]; *P* = .02). *Klebsiella pneumoniae* infection was associated with recurrence in univariate analysis (HR, 1.6 [95% CI, 1.1–2.1]; *P* = .007) but not multivariate analysis (aHR, 1.4 [95% CI, 1.0–1.9]; *P* = .06). Inappropriate initial or definitive antibiotic therapy was not predictive of ESCrE UTI recurrence.

**Conclusions:**

Recurrence of community-onset ESCrE UTI was common and associated with several patient and pathogen-level risk factors. Future studies should evaluate microbial risk factors for recurrence and improve the management of ESCrE UTI.

## Teaser

In this retrospective cohort study of 1,347 patients with community-onset urinary tract infection (UTI) caused by extended spectrum cephalosporin-resistant Enterobacterales (ESCrE), 15% experienced recurrence of ESCrE UTI within 6 months. Independent risk factors for recurrence included neurogenic bladder, prior history of UTI, and fluoroquinolone non-susceptible UTI.

Infections due to extended-spectrum cephalosporin-resistant Enterobacterales (ESCrE), defined as Enterobacterales with nonsusceptibility to third- or fourth-generation cephalosporin antibiotics, have become a leading antimicrobial resistance concern in recent decades due to the global dissemination of extended-spectrum β-lactamase (ESBL) and AmpC β-lactamase–producing organisms [1–4]. According to the United States (US) Centers for Disease Control and Prevention, an estimated 197 400 cases of ESBL-producing Enterobacterales infections and 9100 related deaths occurred among US hospitalized patients in 2017 alone [5].

The threat of ESCrE is not only limited to healthcare settings but increasingly extends into the community. A meta-analysis of ESBL *Escherichia coli* intestinal colonization among healthy individuals reported a global pooled prevalence of 16.5% in 2003–2018, with an increase in pooled prevalence from 2.6% in 2003–2005 to 21.1% in 2015–2018 [4]. Additionally, a study of ESBL-producing bacterial infections in community hospitals in the southeastern US demonstrated a significant rise in community-associated ESBL-producing *E coli* infections from 2009 to 2014, with community-associated infections comprising nearly one-quarter of all ESBL-producing *E coli* infections in the study cohort [3]. Among community-onset ESCrE infections, the majority are urinary tract infections (UTIs), with US studies estimating that cystitis and pyelonephritis cumulatively account for >80% of community-associated infections [2].

Infections caused by ESCrE are of clinical significance because they may be associated with increased risk of mortality, hospital length of stay, and risk of hospital readmission among patients with bacteremia [6–9]. Likewise, community-onset ESCrE UTIs have been associated with increased risk of clinical failure and infection recurrence [10–12]. These poor outcomes are incompletely explained by inappropriate initial antibiotic therapy, suggesting that host and/or microbial factors may play a role in the clinical failures observed with ESCrE UTIs [10].

Despite the increasing epidemiologic and clinical importance of community-onset ESCrE UTIs, little is known about the determinants of unfavorable clinical outcomes among affected patients. Specifically, it is not known whether there are unique predictors for ESCrE UTI recurrence. Here, we evaluate risk factors for infection recurrence among patients with community-onset ESCrE UTIs.

## METHODS

### Study Setting

This retrospective observational cohort study was conducted in the Duke University Health System, which includes a 957-bed academic tertiary care hospital, 2 community hospitals, 11 urgent care facilities, and >140 primary care and subspecialty care clinics in central North Carolina.

### Data Source

Microbiology and antibiotic administration data were obtained from the Duke Antimicrobial Stewardship and Evaluation Team (ASET) database. Demographic variables, laboratory values, *International Classification of Diseases, Tenth Revision* (*ICD-10*) diagnoses, and medication prescription data were obtained from the Duke Enterprise Data Unified Content Explorer (DEDUCE). Both ASET and DEDUCE extract data from the Duke electronic health record (Epic/MaestroCare). All other variables were obtained via direct review of the medical record.

### Study Population

The study population comprised adult patients in the Duke University Health System with community-onset ESCrE UTI between 1 April 2018 and 31 December 2021 who were alive at 14 days after end of antibiotic therapy.

### Study Definitions

Community-onset ESCrE UTI was defined as (1) a urine culture collected in an outpatient setting, emergency department, or within 48 hours of hospital admission with *E coli*, *Klebsiella pneumoniae*, *Klebsiella oxytoca*, or *Proteus mirabilis* testing intermediate or resistant to ceftriaxone or ceftazidime using validated semi-automated methods employed by each study site's respective clinical microbiology laboratory; (2) pyuria (≥10 white blood cells on microscopy or positive leukocyte esterase) on a urinalysis collected within 72 hours before or after urine culture; and (3) administration or prescription of a systemic antibiotic within 7 days after urine culture collection, which was used as a surrogate for symptomatology in the primary analysis due to inconsistent documentation of UTI symptoms in the electronic health record. UTI recurrence was defined as isolation of the same species in a urine culture obtained at least 14 days after and within 180 days following completion of antibiotic therapy for the index infection and satisfaction of the same criteria for infection as in the initial presentation. Inappropriate initial and definitive therapy were defined as nonsusceptibility of the urine isolate to the initial and final antibiotic agents given.

### Statistical Analysis

Data were analyzed in R version 4.1 software (R Foundation, Vienna, Austria) [13]. The time origin was defined as 14 days after end of antibiotic therapy for the index UTI. Patients were right censored upon death or 180 days after end of antibiotic therapy. Unadjusted and adjusted Cox proportional hazards regression was performed; all covariates were determined a priori for inclusion in the adjusted model. The proportional hazards assumption was assessed using Schoenfeld residuals. To explore whether results may have been biased by patients with asymptomatic bacteriuria, a preplanned sensitivity analysis was performed in which the multivariate analysis was restricted to patients with specific signs and/or symptoms of UTI documented by the clinical provider in the medical record during the index infection. Signs and/or symptoms of UTI were defined as lower urinary tract symptoms (eg, dysuria, urinary urgency, hematuria, suprapubic or flank pain) or systemic signs of infection (eg, fever, chills) not attributable to another diagnosis and were adjudicated by infectious diseases–trained physicians (H. L. Z., R. P., J. K.). A post-hoc subgroup analysis was performed to evaluate risk of recurrence among patients with *E coli* UTI only, and a post-hoc exploratory analysis was performed to compare risk of recurrence among patients receiving specific antibiotic agents; adjusted exploratory models included the same covariates as used in the primary models.

Two-tailed *P* values are reported, and an α of .05 was used to define statistical significance. Multiple comparisons adjustments were not performed.

### Patient Consent Statement

This study received approval from the Duke University Institutional Review Board (Pro00111525). A waiver of informed consent was obtained for the study.

## RESULTS

Among 1347 patients with community-onset ESCrE UTI, 202 (15.0%) experienced recurrence in the 6-month follow-up period (Supplementary Figure 1). Cohort characteristics are summarized in Table 1. The emergency department served as the initial site of care for 830 (61.6%) patients. Two hundred and forty-one (17.9%) patients had pyelonephritis including 120 (8.9%) with bacteremia, and 149 (11.1%) patients died between 14 and 180 days following completion of treatment for the index UTI.

**Table 1.** Cohort Characteristics (N = 1347)

Characteristic	No. (%)
Age, y, median (IQR)	68 (54–77)
Male sex	424 (31.5)
Race	
White	733 (54.4)
Black/African American	440 (32.7)
Other/Not reported	174 (12.9)
Ethnicity	
Hispanic/Latino	101 (7.5)
Not Hispanic/Latino	1216 (90.3)
Not reported	30 (2.2)
Comorbidities	
UTI within past year	728 (54.0)
Diabetes mellitus	593 (44.0)
Chronic renal insufficiency	478 (35.5)
Renal transplant	64 (4.8)
Neurogenic bladder	133 (9.9)
BPH or prostate cancer	237 (17.6)
Index UTI characteristics	
Urinary catheter present	112 (8.3)
Urologic stone present	153 (11.4)
Bacteremia or pyelonephritis	241 (17.9)
Pathogen	
*Escherichia coli*	1018 (75.6)
*Klebsiella pneumoniae*	257 (19.1)
*Klebsiella oxytoca*	35 (2.6)
*Proteus mirabilis*	37 (2.8)
Susceptible antibiotics	
Ciprofloxacin (1 not tested)	422 (31.3)
TMP-SMX	509 (37.8)
Nitrofurantoin	970 (72.0)
Piperacillin-tazobactam	1226 (91.0)
Initial antibiotic	
Ceftriaxone	389 (28.9)
Piperacillin-tazobactam	199 (14.8)
Nitrofurantoin	190 (14.1)
Ciprofloxacin	115 (8.5)
Other	454 (33.7)
Inappropriate initial therapy	653 (48.5)
Definitive antibiotic	
Nitrofurantoin	277 (20.6)
Ciprofloxacin	164 (12.2)
Amoxicillin-clavulanate	160 (11.9)
TMP-SMX	142 (10.5)
Other	604 (44.8)
Inappropriate definitive therapy	240 (17.8)
Antibiotic duration, d, median (IQR)	7 (5–10)

Data are presented as No. (%) unless otherwise indicated.

Abbreviations: BPH, benign prostatic hyperplasia; IQR, interquartile range; TMP-SMX, trimethoprim-sulfamethoxazole; UTI, urinary tract infection.

In univariate analysis, risk factors for UTI recurrence included *K pneumoniae* infection (hazard ratio [HR], 1.6 [95% confidence interval {CI}, 1.1–2.1]; *P* = .007), diabetes mellitus (HR, 1.5 [95% CI, 1.1–2.0]; *P* = .004), chronic renal insufficiency (HR, 1.6 [95% CI, 1.2–2.2]; *P* < .001), neurogenic bladder (HR, 2.1 [95% CI, 1.4–3.0]; *P* < .001), history of UTI within 1 year preceding the index ESCrE UTI (HR, 2.8 [95% CI, 2.0–3.8]; *P* < .001), fluoroquinolone nonsusceptibility (HR, 1.6 [95% CI, 1.1–2.2]; *P* = .006), and trimethoprim-sulfamethoxazole nonsusceptibility (HR, 1.4 [95% CI, 1.0–1.8]; *P* = .04) (Table 2).

**Table 2.** Univariate and Multivariate Analyses of Risk Factors for Recurrence of Community-Onset Urinary Tract Infection Caused by Extended-Spectrum Cephalosporin-Resistant Enterobacterales

Candidate Risk Factor	Univariate		Multivariate	
	HR (95% CI)	*P* Value	aHR (95% CI)	*P* Value
Age (per 1-y increase)	1.01 (1.00–1.01)	.26	1.00 (.99–1.01)	.64
Male sex	1.09 (.81–1.46)	.57	0.86 (.55–1.34)	.51
Pathogen (ref: *Escherichia coli*)				
*Klebsiella oxytoca*	1.07 (.44–2.61)	.88	0.98 (.39–2.46)	.97
*Klebsiella pneumoniae*	1.55 (1.13–2.13)	.007	1.37 (.98–1.90)	.06
*Proteus mirabilis*	1.20 (.53–2.73)	.66	1.18 (.52–2.69)	.70
Bacteremia or pyelonephritis	1.23 (.88–1.72)	.24	1.24 (.88–1.76)	.22
Diabetes mellitus	1.49 (1.13–1.97)	.004	1.26 (.94–1.70)	.12
Chronic renal insufficiency	1.64 (1.25–2.17)	<.001	1.29 (.94–1.76)	.11
BPH or prostate cancer	1.08 (.76–1.54)	.67	1.07 (.64–1.79)	.80
Neurogenic bladder	2.07 (1.44–2.96)	<.001	1.77 (1.19–2.65)	.005
Renal transplant	1.48 (.86–2.55)	.16	1.00 (.55–1.79)	.99
UTI within the past year	2.79 (2.03–3.85)	<.001	2.38 (1.70–3.31)	<.001
Inappropriate initial therapy	0.87 (.66–1.15)	.34	1.02 (.75–1.39)	.91
Inappropriate definitive therapy	0.83 (.57–1.22)	.35	0.89 (.58–1.36)	.59
Fluoroquinolone nonsusceptible	1.58 (1.14–2.19)	.006	1.50 (1.07–2.10)	.019
TMP-SMX nonsusceptible	1.36 (1.01–1.82)	.042	1.12 (.82–1.52)	.47

Abbreviations: aHR, adjusted hazard ratio; BPH, benign prostatic hyperplasia; CI, confidence interval; HR, hazard ratio; TMP-SMX, trimethoprim-sulfamethoxazole; UTI, urinary tract infection.

In multivariate analysis, risk factors for UTI recurrence included neurogenic bladder (adjusted HR [aHR], 1.8 [95% CI, 1.2–2.6]; *P* = .005), history of UTI within 1 year (aHR, 2.4 [95% CI, 1.7–3.3]; *P* < .001), and fluoroquinolone nonsusceptibility (aHR, 1.5 [95% CI, 1.1–2.1]; *P* = .02). The association between *K pneumoniae* infection and recurrence was not statistically significant in multivariate analysis (aHR, 1.4 [95% CI, 1.0–1.9]; *P* = .06). Neither inappropriate initial therapy (HR, 0.87 [95% CI, .66–1.15], *P* = .34; aHR, 1.02 [95% CI, .75–1.39], *P* = .91) nor inappropriate definitive therapy (HR, 0.8 [95% CI, .6–1.2], *P* = .35; aHR, 0.9 [95% CI, .6–1.4], *P* = .59) predicted recurrence in univariate or multivariate analysis. Duration of antibiotic therapy was also not associated with recurrence (HR per 1-day increase in duration, 1.0 [95% CI, 1.0–1.0], *P* = .60; aHR, 1.0 [95% CI, 1.0–1.0], *P* = .25).

In a sensitivity analysis restricted to patients with specific signs or symptoms of UTI documented in the medical record (n = 823), *K pneumoniae* infection (aHR, 1.6 [95% CI, 1.0–2.3]; *P* = .03), neurogenic bladder (aHR, 1.9 [95% CI, 1.2–3.1]; *P* = .009), and history of UTI within 1 year (aHR, 2.7 [95% CI, 1.7–2.1]; *P* < .001) were identified as independent risk factors for recurrence (Supplementary Table 1). The association between fluoroquinolone nonsusceptibility and recurrence was not statistically significant in this analysis (aHR, 1.5 [95% CI, 1.0–2.3]; *P* = .06).

In a subgroup analysis restricted to patients with *E coli* UTI, chronic renal insufficiency (aHR, 1.6 [95% CI, 1.1–2.3]; *P* = .017), history of UTI within 1 year (aHR, 2.3 [95% CI, 1.6–3.5]; *P* < .001), and fluoroquinolone nonsusceptibility (aHR, 1.8 [95% CI, 1.2–2.8]; *P* = .009) were independently associated with UTI recurrence (Supplementary Table 2). Neurogenic bladder was not associated with recurrence in this subgroup.

In exploratory analysis, use of doxycycline as the definitive antibiotic (n = 38) was not associated with recurrence compared to ciprofloxacin (n = 164) (HR, 1.4 [95% CI, .5–3.4], *P* = .50; aHR, 1.5 [95% CI, .5–4.1]; *P* = .48). Likewise, use of amoxicillin-clavulanate as the definitive antibiotic (n = 160) was not associated with recurrence compared to ciprofloxacin (HR, 1.3 [95% CI, .7–2.4], *P* = .41; aHR, 1.2 [95% CI, .7–2.3], *P* = .51).

## DISCUSSION

To our knowledge, this study is the first to examine risk factors for recurrence of community-onset ESCrE UTIs. As expected, neurogenic bladder was found to be a strong risk factor for ESCrE UTI recurrence. UTI is a leading cause of morbidity associated with neurogenic bladder, occurring among more than one-third of patients during the first year after diagnosis and accounting for >20% of hospitalizations among patients with neurogenic bladder [14]. Due to frequent healthcare exposure and antibiotic use, patients with neurogenic bladder are also at increased risk of UTI due to ESBL-producing organisms [15, 16]. Our finding that patients with neurogenic bladder who experience an ESCrE UTI are also at particularly high risk of subsequent recurrences further highlights the need to consider these patients as a priority population for developing new strategies to prevent antibiotic-resistant UTIs.

Infection by *K pneumoniae* was found to predict increased risk of ESCrE UTI recurrence in univariate analysis and sensitivity analysis; in our primary multivariate analysis, the effect size estimate was slightly attenuated and below the threshold for statistical significance. One likely explanation for this signal is that host comorbidities that are associated with UTI recurrence, including those that were unmeasured in this study, impact the pathogen distribution pattern in ESCrE UTI. *Klebsiella pneumoniae* is often nosocomially acquired, so a higher prevalence of *K pneumoniae* intestinal carriage, and thus UTI, can be expected among patients with frequent healthcare exposures [17]. Whether there are underlying differences in the clinical course of ESCrE *K pneumoniae* and *E coli* UTIs, independent of host factors, remains to be determined. The existing literature on differences in outcomes of ESBL *K pneumoniae* versus ESBL *E coli* infections is inconclusive. Some studies have reported increased 30-day mortality among ESBL *K pneumoniae* compared to ESBL *E coli* infections [18, 19], while others have shown no difference in outcomes [20]. Future studies are needed to elucidate the role of organism type in outcomes of ESCrE UTIs.

Interestingly, fluoroquinolone nonsusceptibility was a risk factor for ESCrE recurrence, even in multivariate analysis adjusting for appropriateness of the administered antibiotics. On the pathogen level, there is not a clear mechanistic explanation for this association; conversely, fluoroquinolone-resistant uropathogenic *E coli* has been reported to exhibit lower virulence and decreased risk of invasive disease [21, 22]. However, we expect that there are differences in host characteristics among those with fluoroquinolone-susceptible versus nonsusceptible ESCrE UTI. Previous literature has reported that fluoroquinolone-resistant UTI is associated with multiple clinical factors including complicated infection, recent use of antibiotics, and recent hospitalizations, reflecting a population that is overall sicker and with greater comorbidities [23]. Additionally, given that fluoroquinolones represent a preferred oral agent for complicated UTI and stepdown therapy of gram-negative bacteremia, fluoroquinolone resistance may in part reflect prior fluoroquinolone exposure due to complicated UTIs. The clinical outcomes of patients with extended-spectrum cephalosporin and fluoroquinolone co-resistance still warrant further study.

There are several notable findings regarding our cohort. First, recurrence of ESCrE UTI was common, affecting 15% of patients in the 6-month follow-up period. While this statistic is similar to or lower than published incidences of overall UTI recurrence (26%–44% among women and 13% among men in 6–12 months), our study considers only recurrences caused by the same uropathogen as the index infection [24–26]. The rate of overall UTI recurrence in our cohort is therefore likely to be substantially higher than 15%. Second, there was a high prevalence of nonsusceptibility to most antibiotic agents with oral formulations, including 69% nonsusceptibility to fluoroquinolones and 62% nonsusceptibility to trimethoprim-sulfamethoxazole, highlighting the limited therapeutic options for these infections. Third, there was a high prevalence of complicated UTI, with nearly one-fifth of patients experiencing pyelonephritis or bacteremic UTI. However, it is important to note that our study cohort is unlikely to be population-representative, with more than one-half of patients presenting to a hospital as the initial site of care, reflecting a higher severity of illness. Fourth, only approximately one-half of patients in this cohort received an empiric antibiotic agent to which the uropathogen was susceptible, with greater than one-quarter receiving ceftriaxone as the initial antibiotic. This reflects an unmet need to develop and implement strategies that improve the prediction of ESCrE or ESBL UTI at the point of care.

This study has several potential limitations. Given the retrospective nature of our study, our cohort may have included patients with asymptomatic bacteriuria who were inappropriately treated with antibiotics. To address this potential threat to validity, we performed a sensitivity analysis including only patients with documented specific signs or symptoms of UTI, which yielded overall similar findings to the primary analysis. UTI recurrence may have been underascertained among patients who sought care outside of our health system and among those for whom a urine culture was not obtained during the recurrent episode. Comorbidities may have been misclassified if not recorded accurately in the electronic medical record. Outpatient antibiotics may also have been misclassified due to discrepancies between antibiotic prescriptions and those actually taken by the patient. While our definition for UTI recurrence required the uropathogen to be the same species as the index infecting pathogen, we did not collect urinary isolates to determine whether the recurrent episode was caused by the same bacterial strain. Additionally, ESBL phenotypic testing was not routinely performed in our clinical microbiology laboratories, so our study is unable to distinguish between UTIs caused by ESBL-producing versus non-ESBL-producing ESCrE. Finally, this study was performed within a single health system, limiting its generalizability.

In conclusion, we identified several predictors of ESCrE UTI recurrence, including neurogenic bladder, prior UTI, and fluoroquinolone nonsusceptibility. These findings can help to risk stratify patients with ESCrE UTI and identify those who may derive the greatest benefit from early referral to infectious disease and urology subspecialty care. Our study also provides hypothesis-generating data regarding the potential role of pathogen species in ESCrE UTI recurrence. Future well-designed studies are necessary to clarify microbial risk factors for recurrence and to develop effective strategies to decrease recurrence risk among patients with ESCrE UTI.

## Supplementary Material

ofad561_Supplementary_Data

## Supplementary Data

Supplementary materials are available at *Open Forum Infectious Diseases* online. Consisting of data provided by the authors to benefit the reader, the posted materials are not copyedited and are the sole responsibility of the authors, so questions or comments should be addressed to the corresponding author.

## Notes

***Financial support***. H. L. Z. was supported by an Antibacterial Resistance Leadership Group fellowship (National Institute of Allergy and Infectious Diseases award number UM1AI104681).
