# Supplementary material for: Risk Factors for Recurrence of Community-Onset Urinary Tract Infections Caused by Extended-Spectrum Cephalosporin-Resistant Enterobacterales
Source: Open Forum Infect Dis. 2023 Nov 8;10(12):ofad561. doi: 10.1093/ofid/ofad561 (PMC10733196; doi:10.1093/ofid/ofad561)
Supplement: ofad561_Supplementary_Data [file ofad561_supplementary_data.docx]

Supplementary Figure 1. Kaplan-Meier plot of recurrence-free probability among patients with community-onset urinary tract infection caused by extended spectrum cephalosporin-resistant Enterobacterales.


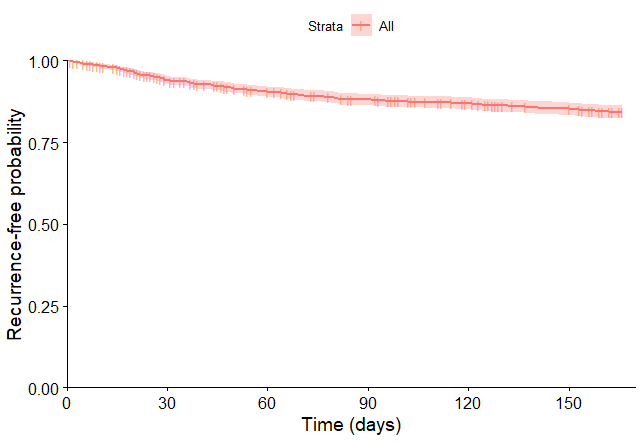


Supplementary Table 1. Multivariate analysis of risk factors for recurrence of community-onset urinary tract infection (UTI) caused by extended spectrum cephalosporin-resistant Enterobacterales among patients with documented signs or symptoms of UTI (N=823).

| Candidate Risk Factor | Adjusted Hazard Ratio (95% Confidence Interval) | p-value |
| --- | --- | --- |
| Age (per 1 year increase) | 1.00 (0.99 – 1.01) | 0.71 |
| Male sex | 0.86 (0.51 – 1.44) | 0.57 |
| Pathogen (ref: E. coli) |  |  |
| *K. oxytoca* | 0.86 (0.26 – 2.78) | 0.80 |
| *K. pneumoniae* | 1.55 (1.05 – 2.29) | 0.028 |
| *P. mirabilis* | 1.08 (0.40 – 2.98) | 0.87 |
| Bacteremia or pyelonephritis | 1.17 (0.80 – 1.70) | 0.41 |
| Diabetes mellitus | 1.30 (0.91 – 1.87) | 0.15 |
| Chronic renal insufficiency | 1.37 (0.93 – 2.01) | 0.11 |
| BPH or prostate cancer | 0.93 (0.49 – 1.73) | 0.81 |
| Neurogenic bladder | 1.89 (1.17 – 3.07) | 0.009 |
| Renal transplant | 1.03 (0.55 – 1.94) | 0.93 |
| UTI within the prior one year | 2.66 (1.74 – 4.07) | <0.001 |
| Inappropriate initial therapy | 1.13 (0.78 – 1.62) | 0.52 |
| Inappropriate definitive therapy | 0.89 (0.53 – 1.53) | 0.69 |
| Quinolone non-susceptible | 1.50 (0.98 – 2.30) | 0.062 |
| TMP-SMX non-susceptible | 1.04 (0.72 – 1.50) | 0.85 |

Abbreviations: aHR = adjusted hazard ratio; BPH = benign prostatic hyperplasia; TMP-SMX = trimethoprim-sulfamethoxazole; UTI = urinary tract infection

Supplementary Table 2. Multivariate analysis of risk factors for recurrence of community-onset urinary tract infection (UTI) caused by extended spectrum cephalosporin-resistant *Escherichia coli* (N=1018).

| Candidate Risk Factor | Adjusted Hazard Ratio (95% Confidence Interval) | p-value |
| --- | --- | --- |
| Age (per 1 year increase) | 1.00 (0.99 – 1.01) | 0.99 |
| Male sex | 1.02 (0.59 – 1.74) | 0.96 |
| Bacteremia or pyelonephritis | 1.04 (0.67 – 1.60) | 0.87 |
| Diabetes mellitus | 1.23 (0.86 – 1.75) | 0.26 |
| Chronic renal insufficiency | 1.58 (1.08 – 2.30) | 0.017 |
| BPH or prostate cancer | 0.97 (0.51 – 1.83) | 0.92 |
| Neurogenic bladder | 1.59 (0.96 – 2.62) | 0.072 |
| Renal transplant | 0.80 (0.36 – 1.78) | 0.59 |
| UTI within the prior one year | 2.35 (1.59 – 3.47) | <0.001 |
| Inappropriate initial therapy | 1.08 (0.74 – 1.57) | 0.69 |
| Inappropriate definitive therapy | 0.85 (0.51 – 1.42) | 0.54 |
| Quinolone non-susceptible | 1.81 (1.16 – 2.83) | 0.009 |
| TMP-SMX non-susceptible | 1.11 (0.78 – 1.58) | 0.56 |

Abbreviations: aHR = adjusted hazard ratio; BPH = benign prostatic hyperplasia; TMP-SMX = trimethoprim-sulfamethoxazole; UTI = urinary tract infection
